# Experimental Infection of Ground Squirrels (*Spermophilus tridecemlineatus*) with Monkeypox Virus

**DOI:** 10.3201/eid1009.040310

**Published:** 2004-09

**Authors:** Robert B. Tesh, Douglas M. Watts, Elena Sbrana, Marina Siirin, Vsevolod L. Popov, Shu-Yuan Xiao

**Affiliations:** * University of Texas Medical Branch, Galveston, Texas, USA

**Keywords:** monkeypox, poxvirus infection, orthopoxvirus, experimental infection, animal model, research

## Abstract

A proposed new small-animal (rodent) model for studying the pathogenesis and treatment of severe orthopoxvirus infections is described. Thirteen-lined ground squirrels (*Spermophilus tridecemlineatus*) were infected intraperitoneally and intranasally with monkeypox virus (MPXV). A fulminant illness developed in all animals, and they died 6–9 days after infection. Virus was cultured from the blood and oropharynx several days before death; at necropsy, all of the organs tested contained relatively high titers of MPXV. The major pathologic findings were in the liver, which showed centrilobular necrosis, steatosis, and basophilic inclusion bodies in hepatocytes. Splenic necrosis was also observed, as well as interstitial inflammation in the lungs. The pathologic features of MPXV in ground squirrels are similar to that described with MPXV in macaques and severe variola (smallpox) virus infection in humans.

## Abstract 2

A new, small-animal model of severe orthopoxvirus infection (monkeypox) is described.

Until last year, human monkeypox was confined to forested areas of central and West Africa, where sporadic epizootics have occurred (*1*). However, in 2003 monkeypox appeared in the United States, and 32 human cases were confirmed during an outbreak that occurred in pet owners in the Midwest (*2**,**3*). Imported African rodents were implicated as the probable source of the outbreak, although the virus also infected other wild animal pets (i.e., prairie dogs) that had contact with them (*4*). On the basis of these reports and earlier studies in Africa (*5**–**7*) that suggest that squirrels and certain other wild rodents might be reservoirs of monkeypox virus (MPXV), we tested the susceptibility of several North American wild rodent species to MPXV infection. We report the results of our studies with the common thirteen-lined ground squirrel, *Spermophilus tridecemlineatus*.

## Materials and Methods

### Animals

Ten adult thirteen-lined ground squirrels (*S. tridecemlineatus*) were used in the experiment. The animals were wild-caught and purchased from a commercial supplier (TLS Research, Bloomington, IL). Ground squirrels were housed individually in filter-bonneted, solid bottom (123-cm^2^ floor area) plastic cages in an isolation room within an animal biosafety level 3 facility. All persons handling the animals had recently received smallpox (vaccinia) vaccination and used appropriate personal protection. Animals were cared for in accordance with the guidelines of the Committee on Care and Use of Laboratory Animals (Institute of Laboratory Animal Resources, National Research Council) under an animal use protocol approved by the Institutional Animal Care and Use Committee at the University of Texas Medical Branch.

### Virus

The strain of MPXV used was designated MPX 2003 and was provided by the Centers for Disease Control and Prevention, Atlanta, Georgia. This virus was originally isolated from a skin lesion from a human monkeypox patient during the 2003 U.S. outbreak (*3*). A stock of the virus was prepared from infected Vero cells; the unsonicated frozen cell lysate was used to infect the rodents and had a titer of 10^6.l^ PFU/mL.

### Virus Assay

Samples for virus assay were stored at –80°C. Before testing, tissue samples were thawed and triturated in sterile Ten Broeck glass tissue grinders in phosphate-buffered saline (PBS), pH 7.4, containing 30% heat-inactivated (56°C for 30 min) fetal bovine serum (FBS) to prepare an approximate 10% (wt/vol) tissue homogenate. After centrifugation at 6,000 rpm for 5 min to clarify the suspension, serial 10-fold dilutions from 10^–1^ to 10^–8^ were prepared in PBS containing 10% FBS. Similar dilutions were made with the blood and throat swab suspensions for virus assay.

Dilutions of the tissue homogenates, blood, and throat swab suspensions were titrated in 24-well cultures of Vero cells; four wells were used for each dilution, as described (*8*). Cultures were incubated at 37°C, and plaques were counted 4–6 days later. Virus titers were defined as the number of PFU per milliliter of sample.

### Experimental Infection of Animals

Ground squirrels were infected by the intraperitoneal (IP) or intranasal (IN) routes. Five squirrels were injected IP with 10^5.1^ PFU of MPX 2003 virus. Five other animals were infected by the IN route; under Halothane (Halocarbon Laboratories, River Edge, NJ) anesthesia, two drops of the stock virus solution containing 10^6.1^ PFU/mL were instilled into each nostril. After infection, all rodents were observed daily for signs of illness; if an animal died, a necropsy was performed, and tissues (liver, spleen, kidney, adrenal, lung, heart, and brain) were taken for histopathologic examinations and virus titration. In some animals, enlarged mesenteric lymph nodes and thymus were also taken. Blood (100 µL from the retroorbital sinus) and an oropharyngeal swab were also taken daily from each animal for virus assay. The whole blood and the swab were expressed in 900 µL of PBS with 10% FBS.

### Histopathologic and Immunohistochemical Methods

At necropsy, tissue samples were taken from the animals and preserved in 10% buffered formalin for 24 to 48 h, followed by storage in 70% ethanol. After fixation, the samples were processed for routine embedding in paraffin. Four- to 5-µm–thick tissue sections were made and stained by the hematoxylin and eosin method (*9*).

Selected tissue sections were also studied immunohistochemically, by using a vaccinia hyperimmune mouse ascetic fluid, at a dilution of 1:100. A mouse-on-mouse IHC-ISO labeling kit (InnoGenex, San Ramon, CA) was used, according to the manufacturer's instructions and a protocol similar to one described (*9*). The primary antibody (a mouse antivaccinia ascitic fluid) was incubated with the section at 4°C for overnight. Tissue sections from two uninfected animals were used as negative controls.

### Transmission Electron Microscopy (EM)

For EM, formalin-fixed tissues were additionally fixed in a mixture of 2.5% formaldehyde, 0.1% glutaraldehyde, 0.03% trinitrophenol, and 0.03% CaCl_2_ in 0.05 mol/L cacodylate buffer, postfixed in 1% OsO_4_ in 0.1 mol/L cacodylate buffer, stained en bloc in 1% uranyl acetate in 0.1 mol/L maleate buffer, dehydrated in ethanol, and embedded in Poly/Bed 812 (Polysciences, Warrington, PA). Ultrathin sections were cut on a Reichert-Leica Ultracut S ultramicrotome and examined in a Philips 201 electron microscope at 60 kV.

## Results

### Clinical Manifestations

Most of the animals became lethargic and anorexic within 4 or 5 days of infection; however, detectable skin lesions, respiratory distress, or other obvious symptoms of disease did not develop in any of the ground squirrels. The five animals (numbers 11–15) infected IP were moribund or dead within 6 or 7 days after infection; death occurred in the IN-infected group (animals 16–20) approximately 2 days later (Table 1). All of the animals were dead within 9 days of infection.

**Table 1:** Results of virus titrations performed on blood (B) and throat swab (TS) samples from monkeypox virus–infected ground squirrels^a^

Animal no.^b^	Sample	Day after infection								
		1	2	3	4	5	6	7	8	9
11	B	0.7^c^	0	1.4	2.9	4.0	D			
	TS	NT	0	0	3.1	5.3				
12	B	0	0	1.0	2.9	3.8	4.8	D		
	TS	NT	0	0	2.2	3.9	5.8			
13	B	0	0	1.2	3.2	4.2	D			
	TS	NT	0	0	3.9	5.8				
14	B	0	0	0.7	2.5	3.9	D			
	TS	NT	0	0	2.9	5.2				
15	B	0	0	0.7	3.0	3.9	D			
	TS	NT	0	0	2.5	6.0				
16	B	0	0	0	0	1.6	2.1	4.2	D	
	TS	NT	0	0	1.7	3.7	4.6	5.3		
17	B	0	0	0	0.7	2.1	2.7	5.0	NT	D
	TS	NT	0	1.7	2.2	4.0	4.9	5.7	6.2	
18	B	0	0	0	0	2.0	2.5	4.3	D	
	TS	NT	0	1.0	1.9	2.9	3.5	4.6		
19	B	0	0	0	0	0	1.9	4.7	4.2	D
	TS	NT	1.8	2.3	3.0	3.9	4.0	5.4	4.9	
20	B	0	0	0	0	1.0	2.5	5.1	4.8	D
	TS	NT	2.5	1.7	2.5	3.7	3.9	3.7	5.4	

^a^NT, not tested; D, animal dead. ^b^Animals 11–15 were infected intraperitoneally; animals 16–20 were infected intranasally. ^c^Virus titer expressed as log_10_ PFU/mL of sample. Values <10^0.7^ were beyond the sensitivity of the assay and were marked as zero.

### Virus Titrations

Table 1 shows the amount of virus detected in daily blood and throat swab samples taken from the infected rodents. Among the IP-infected squirrels, virus was first detected in the blood on day 3; in contrast, in the IN-infected animals, virus was first detected in the oropharynx (throat swab) on days 2 to 4. Virus titers in the two groups of animals were similar and tended to increase with time.

Table 2 gives the results of virus titrations performed on 10% suspensions of liver, spleen, kidney, lung, heart, and brain taken at necropsy from infected animals. The highest MPXV titers were found in the liver and spleen, but relatively large amounts of virus were detected in the other organs as well. The amount of virus present in the various organ groups (i.e., liver or lung) did not appear to be related to the route of infection, a finding that suggests that the MPXV infection in the rodents was disseminated. The lower virus titers in brain and heart, compared to blood, suggest that MPXV did not replicate appreciably in those organs.

**Table 2:** Results of virus titrations performed on 10% organ suspensions of 10 monkeypox virus–infected ground squirrels^a^

Animal no.^b^	Liver	Spleen	Kidney	Lung	Heart	Brain
11	7.5^a^	6.8	4.4	5.9	3.5	2.4
12	7.2	6.4	4.9	6.1	3.8	1.7
13	7.9	6.7	5.3	5.9	4.5	1.7
14	7.5	6.8	5.0	6.1	3.6	2.6
15	7.9	6.8	4.7	6.7	4.2	4.0
16	7.6	6.4	4.7	6.1	4.2	2.5
17	7.4	6.4	4.8	5.5	3.9	2.0
18	7.8	6.4	5.4	6.0	4.2	2.8
19	7.0	6.4	4.1	5.6	4.1	2.1
20	7.8	6.8	4.9	6.1	5.7	1.7

^a^Samples taken at death (necropsy). Virus titer expressed as log_10_ PFU/mL of 10% tissue suspension. ^b^Animals 11–15 were infected intraperitoneally; animals 16–20 were infected intranasally.

### Pathologic Changes and Immunohistochemical Analysis

**Figure 1:**
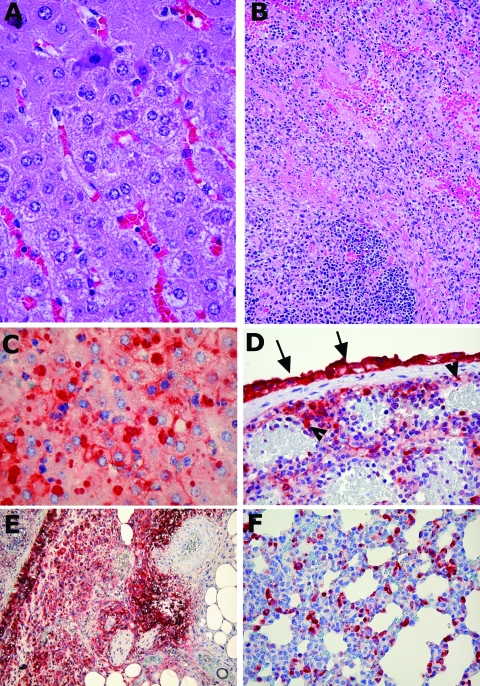
Representative photomicrographs of histologic changes and immunohistochemical staining of tissues from ground squirrels infected with monkeypox virus. A) Liver from a ground squirrel (intranasal infection) showing mild degenerative changes, including early steatosis, and purple-colored viral cytoplasmic inclusion bodies in the hepatocytes (40x objective). B) Spleen from a ground squirrel infected intraperitoneally, showing extensive necrosis (20x objective). C) Liver showing positive antigen staining of the intrahepatocytic inclusion bodies; antigen is present in the cytoplasm and to a lesser extent in cell membranes (40x objective). D) Spleen from a ground squirrel infected intraperitoneally, showing positive antigen staining in the interstitial cells, endothelial cells (arrowheads), and the surface mesothelial cells (arrows) (20x objective). E) Same tissue sample as D, showing the edge of the spleen with antigen-positive mesothelial layer; the adjacent fat and fibrous tissue show necrosis but are also strongly positive for antigen (20x objective). F) Lung from the same animal showing many antigen-positive interstitial cells and pneumocytes (40x objective). A and B, hematoxylin and eosin stain; C–F, immunoperoxidase stain.

In the IP-infected animals (numbers 11–15), considerable centrilobular hepatocytic degeneration or necrosis occurred in the liver. Many hepatocytes, particularly in the areas of degeneration, contained round to oval-shaped basophilic inclusion bodies of various sizes in the cytoplasm. Inflammatory cell infiltration in the lobules was minimal. The portal tracts were normal. Moderate-to-marked necrosis of the spleen was also present in all the animals (Figure 1B). This necrosis was characterized by lymphocytic karyorrhexis in the white pulp, and fibrinoid necrosis, congestion, and endothelial cell swelling in the red pulp, accompanied by cell debris. The lungs showed mild-to-multifocal thickening of the alveolar septa and focal consolidations.

In contrast, the livers of the IN-infected animals (numbers 16–20) exhibited multifocal steatosis; some had a periportal distribution, while others were mainly microvesicular in pattern. In addition, four of the five animals exhibited diffuse hepatocytic necrosis; only one liver (animal 19) had centrilobular necrosis. The characteristic cytoplasmic inclusion bodies were present in all livers (Figure 1A). As observed in the IP-infected animals, moderate-to-severe splenic necrosis occurred. In addition to the variable consolidation and interstitial inflammation in the lungs, some of the IN-infected animals showed necrosis in the peribronchial lymphoid tissue. Lymph nodes from other sites (i.e., mediastinal brown fat) also showed focal necrosis, accompanied by proliferation of immunoblast-like cells, fibroblasts, and macrophages.

Immunohistochemically, no positive staining was observed in control animals. Sections of adrenals, kidneys, and hearts from the infected ground squirrels were also negative. In the liver, most of the larger inclusion bodies stained strongly positive for viral antigen (Figure 1C); however, some of the smaller inclusion bodies were negative. Depending on the severity of the histologic abnormality, this positive staining sometimes involved the surrounding cytoplasm and cytoplasmic membranes. The spleen also stained strongly positive; the intensity generally corresponded to the severity of pathologic changes (Figure 1D). In some animals, the cells lining the surface of the splenic capsule (mesothelial cells) were enlarged and were also strongly positive for viral antigen. In these animals, the positive staining appeared to extend into the superficial zones of the neighboring tissues or organs, such as fat (Figure 1E), pancreas, and adrenal gland, which otherwise were generally negative for viral antigen and lacked pathologic changes. This finding suggests that virus spread directly between adjacent sites when the boundaries (capsules) were broken. Necrotic areas in the perisplenic and periadrenal fat also stained strongly positive.

Viral antigen staining in other organs was less consistent. In the lungs, scattered interstitial cells and a few alveolar pneumocytes were positive (Figure 1F). In the kidneys, sometimes rare mononuclear leukocytes in a few glomeruli were positive. However, these latter positive monocytes probably represented cells in circulation, rather than actual virus replication in the renal tissue.

**Figure 2:**
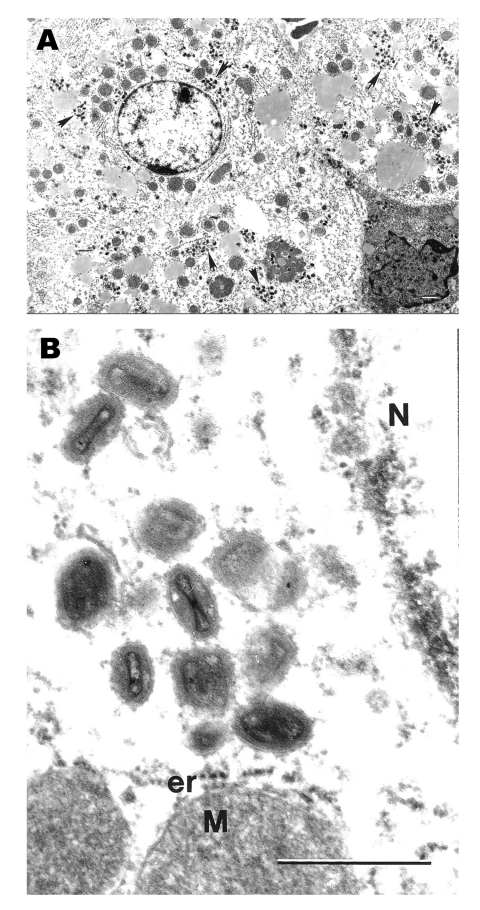
Ultrastructural localization of monkeypox virus in hepatocytes in the liver of a ground squirrel 5 days after infection. A) Hepatocytes contain numerous groups of virions (arrows) in their cytoplasm (bar = 1 µm). B) Magnified area of A, showing typical ultrastructure of monkeypox virus virions and characteristic hepatocyte mitochondria (M) surrounded by cisterns of granular endoplasmic reticulum (er). N, fragment of hepatocyte nucleus; bar = 0.5 µm.

Examination of selected tissues by EM confirmed the results of immunohistochemistry. In ultrathin sections, groups of poxlike virions were readily seen within cytoplasm of infected hepatocytes (Figure 2A and B).

## Discussion

Results of our study indicate that the thirteen-lined ground squirrel is highly susceptible to MPXV. Experimental infection of the animals by both IP and IN routes produced a fulminant uniformly fatal disease. All of the animals were dead by the day 9. The amount and wide distribution of virus in various organs indicate that the infection was disseminated. Initially, the first recovery of MPXV from the blood of IP-infected squirrels and from the oropharynx of IN-infected animals suggested that the pathogenesis of MPXV might be different, depending on the route of infection; however, at necropsy, the amount of virus present in the respective organ systems of the two groups was similar. The histopathology observed at necropsy in the two groups was also similar, although squirrels in the IN-infected group had more hepatic steatosis and pulmonary consolidation. However, this difference may simply be a reflection of the longer incubation period and later death of the IN-infected animals.

In a recent publication, Guarner et al. (*4*) described the histopathologic findings in two sick prairie dogs (*Cynomys* spp.), collected from a pet store during the 2003 monkeypox outbreak in the United States. The abnormal pathologic findings in these two animals were ulcerative lesions on the tongue and conjunctiva and in the lung (bronchioalveolar pneumonia). MPXV was recovered from the lungs of both animals, but only mild inflammation in the liver and reactive hyperplasia in the spleen were found. In another study, we experimentally infected eight prairie dogs with MPXV by the IN and IP routes. Skin or mucosal lesions developed in some of these animals; some of them survived; in general, the survival time was longer and the degree of pathology was less than that observed in the ground squirrels (unpub. data). Although both rodent species are members of the family *Sciuridae*, these observations suggest that MPXV infection in the thirteen-lined ground squirrel is more severe than in prairie dogs.

The fulminant disease and pathology produced in *S. tridecemlineatus* by MPXV are similar to the pathologic findings described in experimentally infected macaques (*10*), which in turn are similar to life-threatening or fatal smallpox (variola virus infection) in humans (*11*). This similarity suggests that ground squirrels might be an excellent small-animal model for studying the pathogenesis and treatment of severe orthopoxvirus infections in people. Concern about potential bioterrorism (*12*) as well as recent reports of zoonotic transmission of poxviruses (*13*) have renewed research interest in these viruses. *S. tridecemlineatus* is abundant in grassland and prairie habitats in the central United States and adjacent regions of Canada (*14*), so supply should be plentiful. Their adult weight (140–252 g), laboratory diet, and cage requirements are similar to those of a large hamster or small guinea pig. Thus, we feel that these animals have considerable value as a laboratory model.
